# New Equations for Hydrostatic Weighing without Head Submersion

**DOI:** 10.3390/jfmk7030070

**Published:** 2022-09-16

**Authors:** Jeff C. Tesch, Panayiotis Papadopoulos, Forrest Dolgener, Grant M. Tinsley

**Affiliations:** 1 EXERTECH, Dresbach, MN 55947, USA; 2 Department of Life and Health Sciences, University of Nicosia, Nicosia 2417, Cyprus; 3 Department of Kinesiology, University of Northern Iowa, Cedar Falls, IA 50614, USA; 4 Department of Kinesiology & Sport Management, Texas Tech University, Lubbock, TX 79409, USA

**Keywords:** underwater weighing, densitometry, hydrodensitometry, body fat percent, body composition

## Abstract

New equations were derived to predict the density of the body (DB) by hydrostatic weighing with the head above water (HW_HAW_). Hydrostatic weighing with the head below water (HW_HBW_) was the criterion for DB measurement in 90 subjects (44 M, 46 F). Head volume by immersion (HV_IMM_) was determined by subtracting the mass in water with the head below water (MW_HBW_) from the mass in water with the head above water (MW_HAW_), with subjects at residual lung volume. Equations were derived for head volume prediction (HV_PRED_) from head measurements and used to correct DB by HW_HAW_. Equations were also derived for HW_HAW_ using direct regression of DB from uncorrected density (with MW_HAW_ in place of MW_HBW_). Prediction equations were validated in 45 additional subjects (21 M, 24 F). Results were evaluated using equivalence testing, linear regression, Bland–Altman plots, and paired *t*-tests. Head girth, face girth, and body mass produced the smallest errors for HV_PRED_. In both M and F validation groups, equivalence (±2% fat by weight) was demonstrated between body fat percent (BF%) by HW_HBW_ and BF% by HW_HAW_ with HV_PRED_. Variance in computer-averaged samples of MW_HAW_ was significantly less (*p* < 0.05) than MW_HBW_. Prediction error was smaller for BF% by HW_HAW_ with HV_PRED_ than for alternative methods. Conclusions: Equivalence between BF% by HW_HBW_ and BF% by HW_HAW_ with HV_PRED_ was demonstrated and differences were not statistically significant. Weight fluctuations were smaller for HW_HAW_ than HW_HBW_.

## 1. Introduction

Health and quality of life have been related to body composition by innumerable scientific studies, as well as anecdotal evidence throughout history. Body composition can be evaluated in numerous ways [1,2], with all methods generally falling within one or more of the five levels defined by Wang et al. [3]: atomic, molecular, cellular, organ/tissue, and whole body. Most common assessment methods utilize the molecular level, which, when implemented as a traditional two-component (2C) model, provides estimates of molecular fat and fat-free mass. The percent of a person’s body weight, which is composed of fat mass (i.e., BF%), has long been recognized to be an important component in the assessment of physical fitness and health. Of the many methods that have been developed to estimate the BF% of living persons, it has long been recognized that measurement of the density of the body (DB) by hydrostatic weighing (HW) is a reliable method [4]; it has been widely used to evaluate body composition [5] and frequently been considered a criterion method against which other indirect methods were validated [6]. The simplest model for HW is the molecular level 2C model by which DB is calculated by assuming a fixed density for the fat and lean components of the body, in order to estimate the BF%. Many equations have been published to estimate BF% from DB using the 2C HW model, both in general and special populations, making it a well-established reference method that has been thoroughly investigated and widely accepted as reliable and valid for the assessment of body composition.

In spite of the high reliability and validity for estimating BF% from DB by HW, a 2013 survey of international sporting organizations from 33 countries found that only 10% reported using HW to assess body composition [7]. This may be due, in part, to the ease of use of alternative methods to estimate BF%, such as skin folds or bioelectrical impedance analysis (BIA). There may also be a perception that the increased complexity of equipment and algorithms implies more valid results for the procedures, such as air displacement plethysmography (ADP) or dual-energy X-ray absorptiometry (DXA). Significant differences, compared to BF% by 2C HW, have been reported for BIA in collegiate wrestlers [8] and for ADP in normal, overweight, and obese groups [9], as well as in lean individuals [10]. Significant differences in BF% have also been reported between four component HW and DXA [11]. While all methods used to estimate BF% have advantages and disadvantages, the simplicity of HW, requiring only a pool of water and accurate scale to weigh an immersed person, make it reasonably accessible and inexpensive, with no need for complex equipment, high levels of expertise, or oversight by radiology personnel. Despite these advantages, a common concern with HW is the need for submersion of the entire body, which could produce discomfort in some participants. Therefore, we thought it would be worthwhile to revisit a previously reported modification of the technique, i.e., HW without head submersion.

In order to calculate DB from HW, the respiratory volume must be accounted for by measurement or estimation. The standard HW procedure [12] requires putting the head completely underwater after a maximal forced exhalation to achieve the smallest possible volume of gas in the airways, which is called the residual volume (RV). However, there is usually no way to be certain that subjects truly exhale maximally during hydrostatic weighing with the head below water (HW_HBW_). In addition, weighing totally immersed subjects can cause procedural difficulties (e.g., turbulence in a small water tank and subjects inhaling water), and it may be stressful for subjects if they are not comfortable under water, particularly at RV.

Hydrostatic weighing with the head above water (HW_HAW_) has been described in several previous investigations. Garrow et al. [4] determined the head volume of 19 female subjects with obesity who were partially immersed in a water tank with the head above water and enclosed in a clear plastic chamber with a microphone–loud speaker system to allow the subject and operator to communicate and requiring pumps to remove water, in order to balance air pressure. Head volume (HV) was determined from pressure fluctuations induced in the air chamber by a separate piston pump and measured by an electronic circuit. A similar principle is used today for whole body ADP, but without the water tank. A much simpler approach was proposed by Donnelly and Smith-Sintek [13], who derived a regression equation (in 40 males) from the length and width measurements of the head to calculate a weight correction for subjects who were partially immersed with the chin and ear lobes at the water line. The immersed subjects were weighed at total lung capacity (TLC), instead of RV, to make them more comfortable and also because HW_HAW_ at RV overloaded their autopsy scale [14]. When their weight correction was applied to an independent group of 11 males, no significant difference in BF% (*p* > 0.05) was found, compared to BF% by 2C HW_HBW_.

In a subsequent investigation Donnelly et al. [15] proposed a different approach, in which they derived regression equations to predict DB directly from the uncorrected “density” obtained by using the weight of the partially-immersed subject in place of the weight of the totally immersed subject. Their modified procedure also included locating a reference mark on the subject’s neck and then raising or lowering the weighing platform to position the mark at the water line during HW. Cross-validation in 20 males (M) and 20 females (F) resulted in no significant differences (*p* > 0.05), compared to BF% by 2C HW_HBW_.

Demura et al. [16] compared BF% by HW_HAW_ to BF% by HW_HBW_ in Japanese subjects (15 M, 15 F) and found significant differences (*p* < 0.01) using either the equation of Donnelly and Smith-Sintek [13] for head weight correction or the equations of Donnelly et al. [15] for DB prediction directly from uncorrected “density”. More recently, Nagao et al. [17] derived new equations to predict HV from head measurements in Japanese subjects who stood on a swing and flexed their knees to raise or lower the head until the chin just touched floating bubble wrap. Head volume was predicted from head measurements and added to the body volume obtained from partial water immersion with the head above water. Using separate validation groups (27M, 56F), they found no significant differences (*p* > 0.05) between BF% by HW_HAW_ and BF% by HW_HBW_ using their equations.

Subjects with obesity often have difficulty with total immersion, due to the buoyancy of body fat, and Evans et al. [14] reported that 25% of patients with morbid obesity could not perform facial immersion. To address this problem, they derived new regression equations to predict DB directly from uncorrected “density”, in the manner of Donnelly et al. [15], but using female subjects with obesity. Heath et al. [18] compared BF% by 2C HW_HBW_ in subjects with obesity and reported that the standard error of estimation (SEE) for BF% by HW_HAW_ using regression on uncorrected “density” was smaller than the SEE of BF% by BIA in both males and females.

Since HW without head submersion seems to have obvious merit, with respect to making the procedure more comfortable, the lack of widespread adoption of the previously published methods for HW_HAW_ may be due to: the complexity of the apparatus for measuring head volume [4]; prediction in only a small number of all male subjects [13]; the need to adjust the depth of the weighing chair in water for each subject [15]; specialized equations developed for subjects with obesity [14,18]; or equations developed using only Japanese subjects [16,17]. Therefore, we proposed to:

1. Derive new equations for HV prediction (HV_PRED_) from simple measurements without special equipment.
2. Compare BF% by HW_HBW_ to both:
  a. BF% by HW_HAW_ with HV_PRED_;
  b. BF% by HW_HAW_ from uncorrected “density”.
3. Compare weight fluctuations in computer samples during HW_HAW_ and HW_HBW_.

## 2. Materials and Methods

Head volume by immersion (HV_IMM_) was determined by weighing subjects who were immersed in water up to the chin and again after complete immersion of the head. By the well-established principle of Archimedes, when a body is partially immersed in water, the difference between the (apparent) mass in water with the head above water (MW_HAW_) and (apparent) mass in water with the head below water (MW_HBW_) is equal to the mass of water displaced by the head (MWDH = MW_HAW_ − MW_HBW_). Dividing MWDH by the density of the water (DW) yields the volume of displaced water, which is also the volume of the head.

Separate equations for HV_PRED_ in males and females were derived from head measurements by multiple regression. The standard equation for calculation of DB [19] was then corrected using HV_PRED_. In this way, DB by HW_HAW_ corrected with a predicted head volume (DB_HAW[HV]_) could be compared to DB by HW_HBW_ (DB_HBW_). RV was chosen as the lung volume for immersion because subjects may have difficulty with total immersion at TLC, due to the buoyancy of the body when the lungs are fully inflated.

New equations to correct HW_HAW_ directly from uncorrected density (without HV_PRED_) were also derived because the equations in previously published methods required raising or lowering the weighing chair [15], which was not possible with a 4 load cell electronic weighing system, or using participants with obesity [14,18].

### 2.1. Participants

Ninety subjects (44 males, 46 females) comprised the experimental (Exp) groups, and 45 additional subjects (21 males, 24 females) comprised the validation (Val) groups. Prior to data collection all participants completed an informed consent form, which was approved by the Institutional Review Board of the University of Northern Iowa. Upon arrival, the participants in both the Exp and Val groups were given a thorough and detailed explanation of the study procedures, and each subject’s permission to participate was obtained. The physical characteristics of the subjects and their immersed weights (partial and total) are described in Table 1.

### 2.2. Anthropometrics

**Figure 1:**
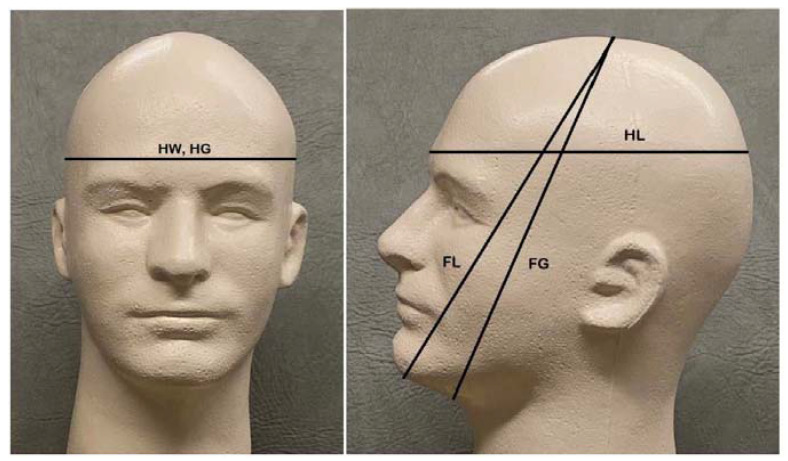
Head measurements. HW, head width: maximum left to right diameter above glabella; HG, head girth: maximum horizontal circumference above glabella; FL, face length: oblique maximum diameter from gnathion to vertex; FG, face girth: oblique maximum circumference under chin to vertex; HL, head length: front to back diameter at the level of the glabella.

Upon arriving in the lab, the height of each subject was measured using a stadiometer. Then, dry body mass in air (MA) was measured in kilograms (kg), by means of a calibrated, beam balance, and weighing scale, with the exact same attire that was to be worn by the subjects inside the water tank. Five head measurements (girths and diameters) were then taken on each subject (Figure 1). The head landmarks were chosen based on the previously published work of Nagao et al. [17]. Head girths were measured with a flexible tape, and head diameters were measured with a spreading caliper with rounded ends (Isokinetics—De Queen, AR, USA). Caliper measurement was verified to the nearest mm with a meter stick. Since girths and diameters in the medial-frontal and the mid-sagittal planes are not directly measurable using a tape measure or caliper, oblique diameter (face length), and oblique circumference (face girth), which were used instead of strictly vertical measurements. All head measurements were taken with the subjects seated on a conventional chair. Measurements were taken by the investigators and two trained student technicians with previous experience in such measurements, who had additional training by the investigators for these particular techniques. The investigators were present at all times during the measurements. To minimize error, participants were asked to keep the mouth closed and not move the jaw. Those with long hair were asked to let the hair down to minimize the effect of hair on the head measurements. This provided clear and direct access to the head landmarks of interest. Head girth and diameter measurements were taken a minimum of twice. Although discrepancies between duplicate measurements were rare, if there was a difference of 0.5 cm or greater, the head measurement was repeated for a third time. The third measurement that was in agreement with one of the first two was recorded as the final value. In the very rare event that three different values were obtained, the entire procedure was repeated until two consistent values were obtained. A single result was recorded for each measurement. No dietary or exercise restrictions or guidelines were provided to the subjects prior to testing.

### 2.3. Hydrostatic Weighing

Subjects wore tight-fitting attire to prevent trapped air while being submerged. Each subject was seated on a weighted chair while immersed in a small, heated, water pool that was specifically designed for HW. The water depth of the pool was approximately 4 feet (1.22 m) deep. Prior to each test, the water temperature in the pool was recorded, and DW was determined using a standard reference table [20]. Immersed weight was measured by means of an electronic weighing system (EXERTECH, Dresbach, MN, USA), which transmitted weight data to a computer and provided a continuous graphic recording. The weighing system was zeroed and then calibrated with a known weight before each testing session. All HW trials were performed at RV after a maximal expiration by the subject. For each weighing trial, a nose clip was affixed to ensure that there was no air leakage through the nasal airways, and the subjects were instructed to:

1. Bend forward until the inferior surface of the chin and ear lobes just touched the water.
2. Exhale maximally with the head positioned as described above and hold the breath at that level.
3. Remain still for two or three seconds with the chin and ear lobes just touching the water.
4. Hold breath at the same level and duck slowly under the water, until the head was completely immersed.
5. Remain still for two or three seconds while completely immersed.
6. Come up for air.

**Figure 2:**
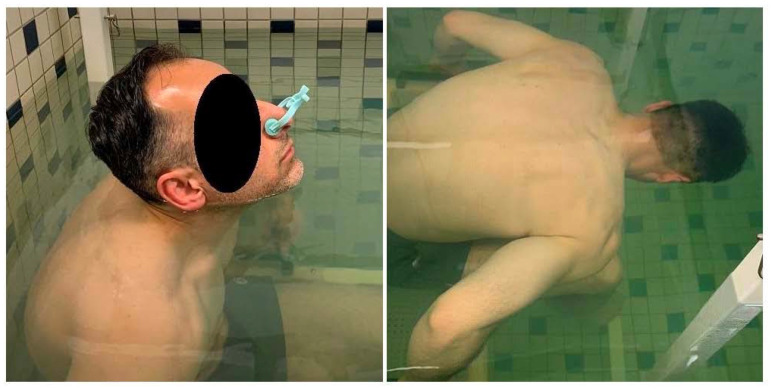
Subject in hydrostatic weighing tank partially immersed with head above water (**left**) and completely immersed with head below water (**right**).

**Figure 3:**
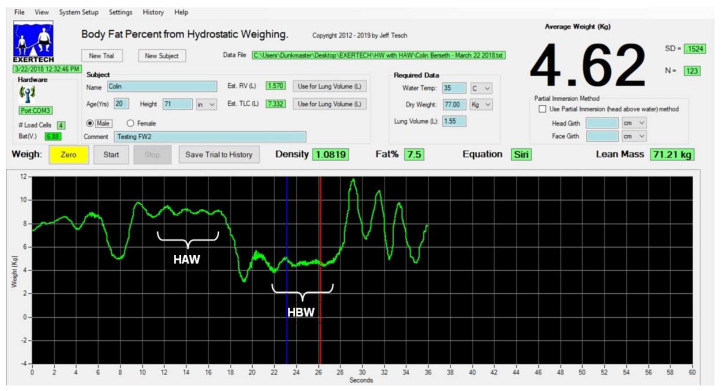
Screen capture of the weighing software showing head above water (HAW) and head below water (HBW) regions of a hydrostatic weighing trial.

The steps above constituted one trial. Figure 2 illustrates the position of the subject above and below the water line. After a short rest to recover from total immersion, subjects repeated the above procedure a minimum of three times. During each trial the weight of the subject was continuously recorded using computer software that was designed specifically for HW (HydroDensity software version HD2, EXERTECH—Dresbach, MN, USA). After each weighing trial was completed, the graph of weight was displayed on the computer screen for selection of the MW_HAW_ and MW_HBW_ segments by means of two movable vertical cursors, which could be positioned using the computer screen pointing device (Figure 3). The average weight, standard deviation (SD) of the weight samples, number of weight samples, DB, and BF% were calculated and displayed by the software each time one of the movable cursors was repositioned along the graph of weight. MW_HAW_ was taken as the average of 100 samples from the graph segment, which showed the smallest weight fluctuations when the subject was partially immersed, as described above. MW_HBW_ was taken as the average of 100 samples from the graph segment, which showed the smallest weight fluctuations when the subject was totally immersed.

In order to achieve consistent HW data, it has been recommended that 10 or more trials should be performed for the immersed weights to approach an asymptotic value [21]. This recommendation was made in lieu of a “practice effect”, which was attributed to the ability of the subjects to expire a greater air volume with successive trials. In the present study, this was not considered to be necessary because subjects were asked to maintain the same level of expiration during the head above water (HAW) and head below water (HBW) portions of each trial. It was, therefore, assumed that the lung volumes under the two conditions were similar enough that 3 trials would suffice for each subject.

Of 135 subjects (75M, 60F) who initially expressed interest in participating in the EXP phase of the study, data from 45 subjects (31M, 14F) were not included in the final analysis. Of 61 subjects (30M, 31F) who were recruited for the VAL phase of the study, data from 16 subjects (9M, 7F) were not included in the final analysis. Reasons for data exclusion included:

1. Preliminary testing data collected before procedures became consistent (EXP group).
2. Failure of potential participants to come for tests at appointed times.
3. Fewer than 3 trials recorded for either MW_HAW_ or MW_HBW_.
4. A difference of more than 0.5 kg in MW_HAW_ between any pair of the 3 trials.
5. A difference of more than 0.5 kg in MW_HBW_ between any pair of the 3 trials.
6. Lack of a stable weight of 3 s duration for either MW_HAW_ or MW_HBW_.

The weight difference (MW_HAW_ − MW_HBW_) upon immersion of the head was taken as the mass of water displaced by the head (MWDH).

[blank line added]

RV was predicted from height and age by means of the equations of Quanjer [22]:

Males: RV (liters) = 1.31∙Height (meters) + 0.022·Age (years) − 1.232(1)

Females: RV (liters) = 1.812·Height (meters) + 0.016·Age (years) − 2.003(2)

The equation of Buskirk [19] was used for calculation of DB_HBW_:

DB_HBW_ = MA·((MA − MW_HBW_)·DW^−1^ − RV − 0.1) ^−1^ (3)

The equation of Brozek et al. [23] was used to estimate BF%:

BF% = 100·(4.57 DB^−1^ − 4.142) (4)

### 2.4. Statistical Analyses

Means, SDs, correlations, linear regression, and *t*-tests were performed independently by two of the authors, one using Excel with the statistical package add-in (Office 365, Microsoft) and another using the software R [24]. In this way, basic statistical calculations and comparisons were cross-checked for accuracy. The level of significance for paired *t*-tests (two-tailed) was set at α = 0.05. To predict HV, multiple regressions were performed using combinations of height, weight, and head measurements. Prediction equations were selected based on the smallest SEE, when compared to the criterion method. The R software was used to assess residuals in the final models for normality and homogeneity of variance, so that the regression model conditions were satisfied. The R software was also used to determine effect size, generate Bland–Altman plots [25], compute confidence intervals for equivalence testing and conduct post hoc analyses of statistical power and minimum sample size. The primary R package used for these analyses was TOSTER. Lin’s concordance correlation coefficient (CCC) [26], including metrics of precision (ρ) and accuracy (Cb), was also calculated. CCC values were interpreted according to McBride’s [27,28] recommendations: almost perfect > 0.99, substantial >0.95 to 0.99, moderate 0.90 to 0.95, and poor <0.90.

Assessments of the normality of the differences between the methods were made by appropriate plots and Shapiro–Wilk normality tests using the R software [24] and deemed to satisfy the requirements of the paired *t*-test for DB and BF% for males, as well as females.

While traditional paired-samples *t*-tests were performed, due to their frequent use and to provide additional information and context, the primary analysis for the validation groups was equivalence testing, which has been deemed a more appropriate method for assessing agreement among measures [29]. Since we could not find any prior research on equivalence testing for predicted *vs* measured HV, equivalence bounds were set at ±0.2 L, which was approximately 5% of the mean head volumes (males, 4.14 L; females 3.66 L) that were measured in the experimental groups. Equivalence bounds for BF% were set at ±2 percent (i.e., absolute fat percent of body weight), which was the mean value from three recently published investigations that used equivalence testing to compare BF% methods [30,31,32]. Non-equivalence was rejected if the 90% confidence interval from two one-sided *t*-tests (TOST) was entirely contained within the equivalence bounds [29].

## 3. Results

### 3.1. Head Volumes

Table 1 shows the results of the head measurements for all groups tested. In the Exp groups, head girth (HG) and face girth (FG) showed the highest individual correlations with MWDH in both the males (HG, r = 0.72; FG, r = 0.63) and females (HG, r = 0.79; FG, r = 0.75). Head length (HL), head width (HW), and face length (FL) showed lower correlations with MWDH in both males (HL, r = 0.50; HW, r = 0.39; FL, r = 0.45) and females (HL, r = 0.53; HW, r = 0.36; FL, r = 0.48). There was also a moderate correlation between MA and MWDH in the females (r = 0.65) and weak correlation in the males (r = 0.37). For all subjects tested (Exp groups + Val groups), the mean HV_IMM_ was 4.19 L for males (*n* = 65) and 3.66 L for females (*n* = 70).

After HW data was collected from the experimental group subjects, HV prediction equations were derived by multiple regression using predictor variables and selected squared transformations (HG, HG^2^, FG, FG^2^, HL, HW, FL, and MA). Based on the prediction errors (SEEs) of candidate multiple regression equations, the following equations were selected to predict head volume:

Males: HV_PRED_ = 0.1294·HG + 0.0299·FG + 0.0055·MA − 5.7506 (5)

Females: HV_PRED_ = 0.1314·HG + 0.0504·FG + 0.0094·MA − 7.3181 (6)

The HV_PRED_ equations were subsequently applied to the Val group subjects. Table 2 presents the means, correlations, and SEEs for all groups tested. The SEE for HV_PRED_ actually decreased in the male Val group (SEE = 0.2333 L), compared to the Exp group (SEE = 0.2596 L). In contrast, in the female Val group, the error for HV_PRED_ (SEE = 0.3425 L) was greater than in the Exp group (SEE = 0.2091 L). This anomaly may be related to the difference seen between the male and female groups, with respect to the changes in correlations between MA (an HV predictor variable) and MWDH with cross-validation. In the males, the correlation between MA and MWDH (Exp, r = 0.37; Val, r = 0.48) increased slightly with cross validation, whereas, in the females, the correlation (Exp, r = 0.65; Val, r = −0.05) decreased substantially, to nearly zero.

Although the SEE did increase with cross-validation in the female subjects, the equivalence between HV_IMM_ and HV_PRED_ was demonstrated in both male and female Val groups (Table 3). The 90% confidence interval for HV_PRED_ in the males (0.001, 0.165 L) and females (−0.163, 0.065 L) were well within the equivalence bounds of ±0.2 L. Post hoc analysis indicated that the observed power for equivalence testing exceeded 0.80 (males: 0.98; females 0.83); correspondingly, the actual sample sizes (21 M, 24 F) exceeded the minimum sample sizes (12 M, 23 F) required for statistical power of 0.8. While equivalence testing was the primary analysis, paired *t*-tests also indicated no significant differences between HV_IMM_ and HV_PRED_ (*p* > 0.05) in either the males or females of the Val groups.

**Figure 4:**
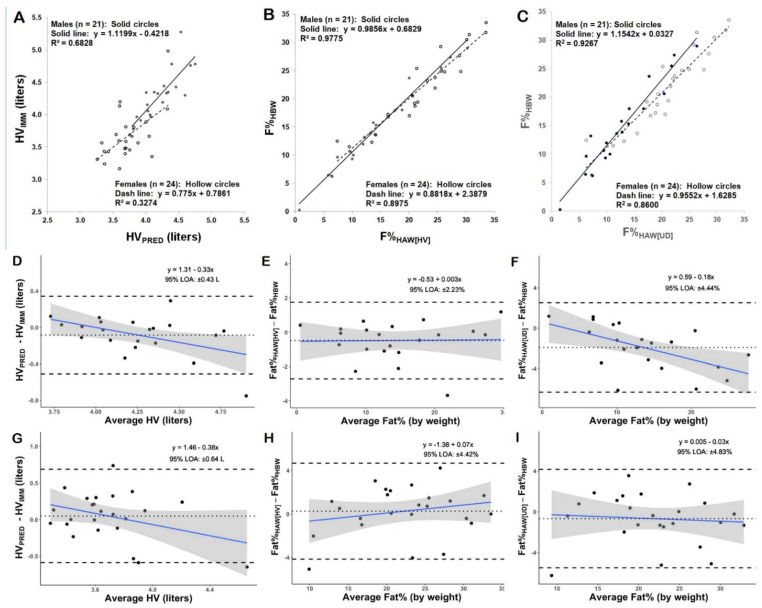
XY and Bland–Altman plots for validation groups. Panels (**A**–**C**) show XY plots for both males and females: (**A**) predicted head volume (HV_PRED_) vs. head volume from immersion (HV_IMM_), (**B**) fat% by head above water immersion with predicted head volume (F%_HAW[HV]_) vs. fat% by head below water immersion (F%_HBW_), and (**C**) fat% by head above water immersion predicted from uncorrected density (F%_HAW[UD]_) vs. fat% by head below water immersion (F%_HBW_). Panels (**D**–**F**) show Bland–Altman plots for the male subjects’ data corresponding to panels (**A**–**C**). Panels (**G**–**I**) show Bland–Altman plots for the female subjects’ data corresponding to panels (**A**–**C**). The solid sloping lines in the Bland–Altman plots are the linear regression lines representing the relationship between the difference between the predicted and criterion values vs. the average of the predicted and criterion values. The shaded regions in the Bland–Altman plots indicate the 95% confidence intervals along the linear regression lines. The horizontal dotted lines indicate the mean of the difference (constant error or mean bias) between methods. The horizontal dashed lines indicate the upper and lower 95% limits of agreement (±1.96SD). The slope of the regression lines in Bland–Altman plots (**E**,**G**–**I**) did not differ from zero (*p* > 0.05), indicating no proportional bias. The slope of the regression lines in plots D and F differed significantly from zero (*p* < 0.05), indicating proportional bias.

The relationship between criterion and predicted values for HV is further illustrated by the XY plots in Figure 4A. In the male subjects, 68% of the variance between HV_IMM_ and HV_PRED_ (R^2^ = 0.6828) was explained by Equation (5). However, in the female subjects, HV_PRED_ by Equation (6) accounted for only 33% of the variance between HV_IMM_ and HV_PRED_ (R^2^ = 0.3274).

Bland–Altman plots were generated to examine the differences between criterion and predicted HV as a function of the average HV (i.e., mean of HV_IMM_ and HV_PRED_). In the male subjects the slope of the regression line (Figure 4D) differed significantly from zero (*p* < 0.05), indicating a proportional bias. In the female subjects, there was also a down-sloping trend line (Figure 4G), but the slope did not differ significantly from zero (*p* > 0.05), suggesting that proportional bias in HV_PRED_ was not present.

### 3.2. Body Density and Percent of Fat

Head volume prediction (HV_PRED_) from either Equation (5) (males) or Equation (6) (females) was subsequently used to modify the standard body density equation (Equation (3)) to predict DB as follows:

DB_HAW(HV)_ = MA·((MA − MW_HAW_)·DW^−1^ + HV_PRED_ − RV − 0.1)^−1^ (7)

In addition to predicting DB_HAW(HV)_ by means of Equation (7), regression equations were also derived to predict DB directly, without a correction for HV. In this case DB was estimated in two steps. First, the standard body density equation (Equation (3)) was used, but with MW_HAW_ in place of MW_HBW_. We refer to the result of this calculation as the uncorrected density (UD):

UD = MA·((MA − MW_HAW_)·DW^−1^ − RV − 0.1)^−1^ (8)

A second step was then performed to predict DB by HW_HAW_ from direct regression of UD (DB_HAW(UD)_), which resulted in the following equations:

Males: DB_HAW(UD)_ = 0.5840·UD + 0.4105 (9)

Females: DB_HAW(UD)_ = 0.5821·UD + 0.4008 (10)

Table 2 summarizes the data for all groups tested, with respect to the means, correlations, and prediction errors (SEE) for DB and BF% by HW, with the head above or below water. Correlations between BF% by HW_HBW_ (BF%_HBW_) and BF% by HW_HAW_ (BF%_HAW_) were higher using HV correction, rather than direct regression of uncorrected density. As a result, prediction errors for BF%_HAW_ using HV correction (BF%_HAW(HV)_) were smaller (males, SEE = 1.16%; females, SEE = 2.14%) than the prediction errors (males, SEE = 2.10%; females, SEE = 2.50%) for the BF%_HAW_ from direct regression of uncorrected density (BF%_HAW(UD)_).

Since the prediction equations were derived from the Exp group, only the Val group data comparisons have been summarized in Table 3. Equivalence between BF%_HBW_ and BF%_HAW(HV)_ was demonstrated by the 90% confidence intervals for both males (0.052% to 0.909%) and females (−1.052% to 0.525%), which were well within the equivalence bounds of ±2% fat. Post hoc analysis for BF%_HBW_ and BF%_HAW(HV)_ indicated that observed power exceeded 0.80 (males: 1.00; females: 0.99); correspondingly, the actual sample sizes (21 M, 24F) exceeded the minimum sample sizes (3 M, 11F) required for a statistical power of 0.8. Equivalence between BF%_HBW_ and BF%_HAW(UD)_ was demonstrated by the 90% confidence interval in the females (−0.197% to 1.527%). However, equivalence between BF%_HBW_ and BF%_HAW(UD)_ was not demonstrated in the males, since the 90% confidence interval (1.063% to 2.767%) extended beyond the upper limit of the equivalence bounds (±2% fat). Post hoc analysis for BF%_HBW_ and BF%_HAW(UD)_ indicated that the observed power for equivalence testing exceeded 0.80 (males: 0.90; females: 0.98); correspondingly, the actual sample sizes (21 M, 24 F) exceeded the minimum sample sizes (11 M, 13 F) required for a statistical power of 0.8.

While equivalence testing was the primary analysis, paired *t*-tests also indicated no significant difference between BF%_HBW_ and BF%_HAW(HV)_ (*p* > 0.05) for either the males or females. No significant difference was observed between BF%_HBW_ and BF%_HAW(UD)_ (*p* > 0.05) in the females. However, in the male subjects, a statistically significant difference between BF%_HBW_ and BF%_HAW(UD)_ (*p* < 0.05) was observed.

The relationships between the criterion and predicted values for BF% are further illustrated by the XY plots in Figure 4. Regression analysis in Figure 4B shows that HW_HAW_ with HV_PRED_ accounted for 98% of the variance (R^2^ = 0.9775) between BF%_HBW_ and BF%_HAW(HV)_ in the male subjects, and 90% of the variance (R^2^ = 0.8975) in the female subjects. In Figure 4C regression analysis showed that the prediction equations for direct regression of uncorrected density explained 93% of the variance (R^2^ = 0.9267) between BF%_HBW_ and BF%_HAW(UD)_ in the male subjects, and 86% of the variance (R^2^ = 0.8600) in the female subjects.

Bland–Altman plots in Figure 4 show the relationships between criterion and predicted BF% differences as a function of the average BF% (i.e., mean of criterion and prediction). In the male subjects, the slope of the regression line (Figure 4E) did not differ significantly from zero (*p* > 0.05), indicating no proportional bias between BF%_HBW_ and BF%_HAW(HV)_. In contrast, the slope of the regression line in Figure 4F (males) did differ significantly from zero (*p* < 0.05), indicating proportional bias between BF%_HBW_ and BF%_HAW(UD)_. In the female subjects, the slopes of the regression lines in both Figure 4H and Figure 4I did not differ significantly from zero (*p* > 0.05), indicating no proportional bias between either BF%_HBW_ and BF%_HAW(HV)_ or BF%_HBW_ and BF%_HAW(UD)_.

We also examined (in the Val groups only) the SD of the 100 data points (approximately 2.5 s of weighing), which were used to calculate the average weight for each trial of MW_HAW_ and MW_HBW_. Paired *t*-tests showed significant differences (*p* < 0.05) for weight fluctuations in both males (SD of MW_HAW_ samples = 0.311 kg; SD of MW_HBW_ samples = 0.396 kg) and females (SD of MW_HAW_ samples = 0.223 kg; SD of MW_HBW_ samples = 0.300 kg).

## 4. Discussion

The present study represents a thorough investigation into the utility of HW with the head above water for body composition assessment. While select prior studies have indicated the potential of this procedure [4,13,14,15,16,17,18], the present work extends these preliminary findings through rigorous methods and thorough analysis. A major finding of the present study is that body fat from HW with the head above water, using head volume predicted from head girth, face girth, and body mass, is statistically equivalent to traditional HW with the head fully submerged. Furthermore, mass measurements were more stable during HW with the head above water, as compared to traditional HW, indicating potential advantages to this procedure beyond clear benefits for participant comfort.

Subjects who completely immerse the head underwater after a maximum forced exhalation tend to do several things that negatively impact the HW procedure. In anticipation of impending breathlessness, they may not exhale completely to RV. While RV is most commonly recommended for HW because it is the smallest lung volume and, therefore, least affected by hydrostatic pressure, exhaling to the point of RV during total immersion HW is a novel and sometimes impossible technique to master for many individuals [33]. This is a common source of error because most HW data is based on either an estimated or previously measured RV. Having the head above water during HW reduces anxiety because subjects have immediate access to air [13]. Giving instructions and coaching a subject throughout the HW procedure is also much easier when the subject’s head is above the water. HW by total immersion at RV also encourages subjects to duck underwater quickly, in order to minimize the time for breath holding, which can create water turbulence in a small pool or HW tank, resulting in scale perturbations that make accurate weighing more difficult, particularly when using a spring scale. Subjects move less and more slowly when HW is performed without head submersion. Furthermore, our data showed that scale fluctuations were significantly lower (*p* < 0.05) during HW_HAW_ than during HW_HBW_. For these reasons, we recommend the use of HW with the head above water, as a viable, if not superior, alternative to traditional HW with full submersion.

Previous research also provided preliminary support for HW without full head submersion. In their pilot study of HW without head submersion in 40 male subjects, Donnelly and Smith-Sintek [13] found SEE = 269.92 g for the (weight) correction factor. Based on the estimated water density for their reported water temperatures (between 32° and 34° C), the equivalent head volume would be about 0.271 L. In our investigation, HV_PRED_ in the male subjects resulted in similar, but slightly smaller, prediction errors (Exp group, SEE = 0.2596 L; Val group, SEE = 0.2333 L). This difference may be due to the fact that Donnelly and Smith-Sintek [13] used head width and head length to calculate their correction factor, whereas we found that head girths provided higher correlations with the mass of water displaced by the head. On the other hand, Nagao et al. [17] reported smaller errors for head volume prediction (SEE = 0.193 L for both males and females) in their Japanese subjects, with mean head volumes (males, 4.31 L; females, 3.74 L) that were very similar to the mean head volumes (from immersion) in our Val group subjects (males, 4.30 L; females, 3.67 L).

Using uncorrected density to predict DB from regression equations, Donnelly et al. [15] reported prediction errors for their experimental groups (males, SEE = 0.0067 g/mL; females, SEE = 0.0061g/mL) that were approximately twice as large as the prediction errors for DB_HAW[HV]_ in our Exp groups (males, SEE = 0.0035 g/mL; females, SEE = 0.0030 g/mL). Prediction errors for DB from uncorrected density in their cross-validation groups (males, SEE = 0.0043 g/mL; females, SEE = 0.0084 g/mL) were also larger than we found in our Val groups (males, SEE = 0.0029 g/mL; females, SEE = 0.0052 g/mL) via Equation (9) (males) or Equation (10) (females).

An extensive comparison of methods other than HW for estimating BF% is beyond the scope of this investigation. However, in order to provide some context for our results, the BF% prediction error (SEE) in the present investigation was compared with 12 other investigations that also used 2C HW_HBW_, as the reference method [8,13,15,17,34,35,36,37,38,39,40,41] (Table 4). Although it is not a comprehensive review of SEE for BF% estimation, the data represent typical errors in estimating BF% by various methods reported in earlier investigations.

These data indicate that the method with the largest prediction error was BIA (males, mean SEE = 3.7%; females, mean SEE = 4.0%). The mean SEE of BF% for the four reports in male subjects using ADP was 2.5%, which was more than twice as large as we found for HW_HAW[HV]_ in males (SEE = 1.2%). From the three reports in female subjects of SEE for BF% by ADP, the mean was 3.2%, which is also larger than we found for BF% by HW_HAW[HV]_ in females (SEE = 2.1%). Comparisons with prediction errors from ADP are particularly relevant to our investigation, since ADP and HW_HAW_ both use DB to estimate BF%.

Errors in BF% by DXA are difficult to compare for a number of reasons. Manufacturers have used different detection, calibration, and analysis techniques, which vary with the instrument model, mode of data collection, and software version [42]. In addition, it is difficult to find published data that reports the SEE for BF% by DXA using BF% by 2C HW_HBW_ as the criterion. This is partly due to the fact that studies that reported BF% by both DXA and 2C HW_HBW_ were typically validated against BF% by multi-component models incorporating HW_HBW_. DXA has also been embraced by some [43] as the reference method for BF% estimation in place of HW_HBW_. In any case, our purpose was not to address the absolute accuracy of BF% estimation, but rather to examine SEE of BF% prediction using BF% by 2C HW_HBW_ as the reference method.

Based on the methods, we compared for male subjects, the smallest prediction error for BF% (SEE = 1.2%) was found by HW_HAW[HV]_ in the present investigation. The smallest SEE for BF% in female subjects (SEE = 1.9%) was found in the study of Nagao et al. [17], which also used equations to estimate HV. These results support previous studies of BF% by HW_HAW_, which found smaller SEEs with HV_PRED_ than with prediction from uncorrected density.

It should be noted that the SEE values in Table 4 are to provide general context for the SEE values observed in the present study, but do not represent a comprehensive, systematic investigation into all reported SEE values. Finally, procedural overlap for BF% estimation from HW_HBW_ and HW_HAW_ is important to consider.

Limitations of the present work include a sole focus on hydrostatic weighing, without consideration of other common body composition assessment methods. In future research, comparisons, including hydrostatic weighing without head submersion, alongside other commonly used methods, can help establish the comparative validity and reliability within the same subjects. Additionally, all subjects in the present study ranged from 18 to 36 years. As such, additional research in youth, middle-aged, and older individuals may help inform the utility of hydrostatic weighing with the head above water. This may be particularly important, due to the difficulties or impracticalities associated with traditional hydrostatic weighing procedures in children and older adults. Finally, the paucity of recent research on hydrostatic weighing with the head above water, beyond the present investigation, indicates the need for continued research on the performance and implementation of this technique.

## 5. Conclusions

Hydrostatic weighing is a well-described and well-accepted method of body composition assessment. HW_HAW_ with HV_PRED_ alleviates many procedural difficulties reported for HW_HBW_. The practicality of HW_HAW_ with HV_PRED_ is also noteworthy. Head girth measurements are painless, non-threatening, require only a tape measure, and can be performed quickly by non-experts with very little training. HW_HAW_ for BF% estimation is convenient and affordable, requiring only a spring scale or electronic weighing system. The procedure can be performed in almost any swimming pool, and calibration requires only an accurate test weight. Without the need for head submersion, HW_HAW_ offers a more pleasant experience than HW_HBW_ for subjects of all ages and at all levels of BF%. Therefore, we believe that further investigations are warranted to improve HV_PRED_ by using larger and more diverse sample populations to develop equations that could be more generally applied in evaluating the effectiveness of exercise and dietary programs, with respect to BF% monitoring.

## Figures and Tables

**Table 1:** Physical measurements and correlations with mass of water displaced by the head for experimental and validation groups.

	Males (Exp: *n* = 44, Val: *n* = 21)						Females (Exp: *n* = 46, Val: *n* = 24)					
	Mean ± SD		Range		r		Mean ± SD		Range		r	
	Exp	Val	Exp	Val	Exp	Val	Exp	Val	Exp	Val	Exp	Val
Physical data												
Age (years)	21.6 ± 3.3	22.1 ± 3.2	18–36	19–35	0.10	−0.18	20.4 ± 1.2	20.5 ± 1.6	19–23	18–26	0.05	0.07
Height (cm)	182 ± 10	177 ± 8	166–210	161–189	0.22	0.49	168 ± 8	166 ± 7	152–185	144–175	0.49	0.05
Weight (kg)	83.3 ± 10.6	81.8 ± 13.5	64.7–116.8	62.5–118.2	0.37	0.48	65.2 ± 10.8	65.9 ± 8.2	41.0–96.6	51.6–79.8	0.65	−0.05
Girths (cm)												
Head girth	57.7 ± 1.7	58.1 ± 1.5	55.0–62.0	55.5–61.0	0.72	0.77	55.5 ± 1.4	55.6 ± 1.7	51.5–58.5	53.0–60.0	0.79	0.58
Face girth	65.4 ± 2.0	66.3 ± 2.0	61.0–72.0	63.5–71.0	0.63	0.83	61.0 ± 1.9	61.8 ± 1.7	54.5–64.0	57.0–64.0	0.75	0.48
Diameters (cm)												
Head length	19.6 ± 0.9	*	17.0–21.5	*	0.50	*	18.9 ± 0.7	*	16.0–20.0	*	0.53	*
Head width	15.3 ± 0.6	*	14.0–17.0	*	0.39	*	14.6 ± 0.5	*	13.5–16.0	*	0.36	*
Face length	25.2 ± 0.8	*	23.0–26.5	*	0.45	*	23.7 ± 0.9	*	21.5–25.5	*	0.48	*
Weights (kg)												
MW_HAW_	8.51 ± 1.01	7.97 ± 1.10	6.22–10.92	5.47–9.95	0.43	0.36	5.76 ± 0.94	5.50 ± 0.80	3.35–8.29	4.04–7.66	0.70	0.52
MW_HBW_	4.39 ± 0.91	3.70 ± 1.03	2.33–6.57	1.52–5.39	0.06	0.02	2.13 ± 0.73	1.86 ± 0.68	0.60–3.86	0.21–3.12	0.37	0.04
MWDH	4.11 ± 0.38	4.27 ± 0.38	2.94–5.11	3.43–5.38	1.00	1.00	3.64 ± 0.39	3.65 ± 0.39	2.47–4.66	3.08–5.35	1.00	1.00

Exp, experimental group; Val, validation group; MW_HAW_, mass in water with head above water; MW_HBW_, mass in water with head below water. MWDH, mass of water displaced by the head upon water immersion; r, Pearson correlation with MWDH. * Not measured in validation group.

**Table 2:** Means, correlations, and standard errors for predicted head volume, body density, and fat percent.

	Mean ± SD		CCC Analysis						SEE	
			CCC		*ρ*		Cb			
Males (Exp: *n* = 44; Val: *n* = 21)	Exp	Val	Exp	Val	Exp	Val	Exp	Val	Exp	Val
Head volume (liters)										
HV_IMM_ (criterion)	4.14 ± 0.38	4.30 ± 0.38								
HV_PRED_	4.14 ± 0.29	4.21 ± 0.28	0.72	0.76	0.75	0.83	0.96	0.93	0.2596	0.2333
Body density (g·ml^−1^)										
DB_HBW_ (criterion)	1.0727 ± 0.0102	1.0662 ± 0.0187								
DB_HAW[HV]_	1.0727 ± 0.0104	1.0674 ± 0.0187	0.94	0.99	0.94	0.99	1.00	1.00	0.0035	0.0029
DB_HAW[UD]_	1.0727 ± 0.0083	1.0709 ± 0.0157	0.79	0.91	0.81	0.96	0.98	0.95	0.0061	0.0052
Fat percent (by weight)										
BF%_HBW_ (criterion)	11.85 ± 4.08	14.55 ± 7.56								
BF%_HAW[HV]_	11.85 ± 4.14	14.07 ± 7.58	0.94	0.99	0.94	0.99	1.00	1.00	1.38	1.16
BF%_HAW[UD]_	11.85 ± 3.30	12.63 ± 6.30	0.79	0.91	0.81	0.96	0.98	0.95	2.41	2.10
**Females (Exp: *n* = 46; Val: *n* = 24)**										
Head volume (liters)										
HV_IMM_ (criterion)	3.66 ± 0.39	3.67 ± 0.39								
HV_PRED_	3.66 ± 0.33	3.72 ± 0.29	0.84	0.54	0.85	0.57	0.99	0.95	0.2091	0.3425
Body density (g·ml^−1^)										
DB_HBW_ (criterion)	1.0528 ± 0.0134	1.0475 ± 0.0157								
DB_HAW[HV]_	1.0528 ± 0.0147	1.0469 ± 0.0170	0.97	0.94	0.97	0.95	1.00	1.00	0.0030	0.0052
DB_HAW[UD]_	1.0528 ± 0.0124	1.0491 ± 0.0154	0.92	0.92	0.93	0.93	0.99	0.99	0.0051	0.0061
Fat percent (by weight)										
BF%_HBW_ (criterion)	19.96 ± 5.56	22.16 ± 6.54								
BF%_HAW[HV]_	19.95 ± 6.08	22.43 ± 7.03	0.97	0.94	0.98	0.95	1.00	1.00	1.25	2.14
BF%_HAW[UD]_	19.95 ± 5.13	21.50 ± 6.35	0.92	0.92	0.93	0.93	1.00	0.99	2.11	2.50

Exp, experimental group; Val, validation group; CCC: Lin’s concordance correlation coefficient; ***ρ***, precision, as indicated by Pearson’s correlation (r); Cb, accuracy based on the bias correction factor; SEE, standard error of estimation; HV_IMM_, head volume by immersion; HV_PRED_, head volume predicted; DB_HBW_, density of the body from hydrostatic weighing with the head below water; DB_HAW[HV]_, density of the body from hydrostatic weighing with the head above water corrected for HV_PRED_; DB_HAW[UD]_, density of the body from hydrostatic weighing with the head above water predicted from uncorrected density; BF%_HBW_, fat percent from DB_HBW_; BF%_HAW[HV]_, fat percent from DB_HAW[HV]_; BF%_HAW[UD]_, fat percent from DB_HAW[UD]_.

**Table 3:** Equivalence tests and *t*-tests for predicted head volume and body fat percent.

Comparison:	HV_IMM_, HV_PRED_	BF%_HBW_, BF%_HAW[HV]_	BF%_HBW_, BF%_HAW[UD]_
**Males (*n* = 21)**			
Equivalence bounds	±0.2 L	±2.0%	±2.0%
90% CI (LL, UL)	(0.001, 0.165)	(0.052, 0.909)	(1.063, 2.767)
Equivalence?	Yes	Yes	No
Power	0.99	1.00	0.98
Minimum sample size ^1^	10	3	11
t (*p*)	1.75 (*p* = 0.095)	1.93 (*p* = 0.067)	3.87 (*p* = 0.001) *
**Females (*n* = 24)**			
Equivalence bounds	±0.2 L	±2.0%	±2.0%
90% CI (LL, UL)	(−0.163, 0.065)	(−1.052, 0.525)	(−0.197, 1.527)
Equivalence?	Yes	Yes	Yes
Power	0.83	0.99	0.98
Minimum sample size ^1^	23	11	13
t (*p*)	0.74 (*p* = 0.466)	0.57 (*p* = 0.572)	1.32 (*p* = 0.199)

HV_IMM_, head volume by immersion; HV_PRED_, head volume predicted; BF%_HBW_, body fat percent by HW with head below water; BF%_HAW[HV]_, body fat percent by HW with head above water corrected with HV_PRED_; BF%_HAW[UD]_, body fat percent with head above water predicted from uncorrected density; CI, confidence interval; LL, lower limit; UL, upper limit; t statistic for paired *t*-test. ^1^ For 80% power, α = 0.05. * *p* < 0.05.

**Table 4:** Standard errors for estimation of body fatness from various methods, compared to hydrostatic weighing.

Investigation	N	Age ± SD	Criterion BF%	Estimated BF%	SEE	Method
**Males**						
Biaggi et al., 1999 [38]	23	33.3 ± 8.7	21.5	20.2	3.1	ADP
Claros et al., 2005 [34]	40	12.4 ± 1.3	18.7	18.4	3.0	ADP
Dixon et al., 2005 [8]	25	19.2 ± 1.2	14.5	13.8	1.7	ADP
Utter et al., 2003 [41]	66	20.2 ± 2.0	11.3	11.0	2.1	ADP
Dixon et al., 2005 [8]	25	19.2 ± 1.2	14.5	12.3	3.6	BIA
Jackson et al., 1988 [35]	50	36.7 ± 9.5	19.1	16.7	4.6	BIA
Lukaski et al., 1986 [36]	47	26.9 ± 8.0	16.2	NR	3.0	BIA
Clark et al., 1993 [39]	35	39.1 ± 14.0	17.4	21.3	3.0	DXA
Wellens et al., 1994 [37]	50	39.9 ± 13.7	22.5	21.7	2.3 *	DXA
Donnelly et al., 1988 [15]	20	NR	15.9	15.9	1.6	HW_HAW[UD]_
Present investigation	21	22.1 ± 3.2	14.6	12.6	2.1	HW_HAW[UD]_
Donnelly and Sintek, 1986 [13]	11	NR	NR	NR	1.4	HW_HAW[HV]_
Nagao et al., 2008 [17]	27	21.8 ± 5.1	13.6	NR	1.3	HW_HAW[HV]_
Present investigation	21	22.1 ± 3.2	14.6	14.1	1.2	HW_HAW[HV]_
**Females**						
Biaggi et al., 1999 [38]	24	30.7 ± 7.2	28.6	29.7	2.5	ADP
Claros et al., 2005 [34]	26	12.0 ± 1.9	20.4	18.9	3.8	ADP
Vescovi et al., 2002 [40]	80	20.2 ± 1.5	19.4	21.2	3.3	ADP
Jackson et al., 1988 [35]	82	28.5 ± 5.7	20.2	22.8	5.0	BIA
Lukaski et al., 1986 [36]	67	27.0 ± 6.4	25.1	NR	3.1	BIA
Wellens et al., 1994 [37]	78	42.5 ± 13.7	33.2	34.6	3.2 *	DXA
Donnelly et al., 1988 [15]	20	NR	21.5	22.2	3.5	HW_HAW[UD]_
Present investigation	24	20.5 ± 1.6	22.2	21.5	2.5	HW_HAW[UD]_
Nagao et al., 2008 [17]	56	20.3 ± 1.8	20.1	NR	1.9	HW_HAW[HV]_
Present investigation	24	20.5 ± 1.6	22.2	22.4	2.1	HW_HAW[HV]_

N, number of subjects; SD, standard deviation; criterion BF%, percent of body fat from (two-compartment) density obtained using total immersion hydrostatic weighing; Estimated BF%, percent of body fat using the specified method; SEE (F%), standard error of estimation of percent of body fat; ADP, air displacement plethysmography; BIA, bioelectrical impedance analysis; DXA, dual electron x-ray absorptiometry; HW_HAW[HV]_, hydrostatic weighing with head above water using density corrected with head volume prediction; HW_HAW[UD]_, hydrostatic weighing with head above water using density predicted from uncorrected density; NR, not reported; * SEE was reported as root mean square error.

## Data Availability

Data requests will be reviewed by the corresponding author.
